# Selections and Abstracts

**Published:** 1911-12

**Authors:** 


### SELECTIONS AND ABSTRACTS

---

### CLINICAL SOCIETY OF NEW YORK POLYCLINIC MEDICAL SCHOOL AND HOSPITAL.

### MEETING OCT. 2ND, 1911.

Dr. T. H. Morgan showed a typical case of cancer of the stomach, in a patient 73 years of age, a laborer, in good health up to three months ago. He first complained of loss of appetite, then loss of flesh; later pain and distress in the stomach, after taking food, with periodical attacks of vomiting every 24, 36 and 48 hours. He was sent to the Hospital and given a test meal. Dr. Jeffries examined the stomach contents, found free hydrochloric acid, Boas bacillus, etc., etc. The patient soon had coffee ground vomit and tarry stools. Marked cachexia. Physical examination showed thickening near pylorus on lesser curvature. Vomiting became more frequent after admission to hospital.

Dr. Albert Morrow advised a gastro-enterostomy in which Drs. Lynch and Sinclair concurred. The patient, however, refused operation. The case was shown as being a typical one of Carcinoma of Stomach.

A Specimen of Cancer of the Stomach was presented by Dr. Albert Morrow. The specimen was removed from a patient 60 years of age; the general character of the symptoms being the same as in Dr. Morgan's case. Vomiting constant; emaciation extreme. The tumor was distinctly felt in the region of the umbilicus, and an exploratory incision was made under local anaesthesia, hyoscine and morphine being given before the operation. The patient lived only three days, but retained food and did not vomit after the operation. She might have recovered but was in extreme condition of exhaustion when taken for operation.

Dr. Hays called attention to the frequent presence of extensive carcinoma, without positive symptoms, until the disease is incurable.

Dr. Jeffries said that the blood in Carcinoma closely resembled that of pernicious anaemia, and at times only an expert could differentiate it. The case showed secondary anaemia with little or no leucocytosis. The stomach contents showed no evidenes of pieces of tumor and there was a total absence of free hydrochloric acid. Pepsin was present.

Dr. Albert Morrow stated that cases of scirrhus chance of the breast might live many years, in spite of considerable emaciation.

Dr. Maurice Packard presented an interesting case of pigmentation of the face, in a man 72 years of age, suggesting Addison's Disease.

Angio-Sarcoma of the Testicle, presented by Dr. Albert Morrow. Dr. Morrow said that he had operated on the patient for Angio-Sarcoma of the left testicle, which he removed. The patient was 45 years of age, and had an undescended testicle of the left side, which came down at the age of seven. Fifteen years ago the same testicle become swollen from an injury, but subsided under hot applications. Twelve years later a hydrocele developed on the same side. This was frequently tapped. A year ago the hydrocele seemed larger and was so annoying that the patient decided to have a radical operation. On examination

Dr. Morrow found apparently two sacs, the lower one hard and elastic. He removed some fluid and thought that the former injections of carbolic acid might have caused the thickening of the sac. On exposing sac, was surprised to find a great number of blood vessels in the sac, and also a great deal of pain present. Upon cutting into hydrocele, a solid, firm mass was found, resembling a blood clot. Further examination revealed a soft growth. Patient was informed that the growth was probably malignant, and consented to have it removed. Pathological examination showed an angio-sarcoma of the testicle. Patient is doing well.

Several interesting points were emphasized. 1. Undescended Testicles are very apt to undergo sarcomatous change. 2. The history of injury and the question as to whether the constant injection of carbolic acid, during previous treatment had anything to do with the condition? The prognosis was very uncertain. Angio-sarcoma may be less malignant than other forms; most cases however are very malignant. Coley's serum was worth trying.

Dr. Sinclair said he had injected many cases of hydrocele with pure carbolic acid, and had never seen a serious case from it, or any evidence of sarcomatous change. He had experience with Coley's serum in only one case. A year after using the serum the patient had bronchitis. He found a consolidation in one lung and a pecuilar cough. The patient did not improve and died suddenly. Autopsy showed sarcomatous material in the lungs, peritoneum and abdominal wall. The case was one of general sarcomatous recurrence.

Dr. Doran had been working in sarcoma with amylopsin and trypsin. He gave hypodermically twenty minims of each every other day for about six weeks, gradually working up to 60 minims of amylopsin and trypsin. With care he avoided abscesses.

Dr. Joseph Taylor reported a case of Carcinoma which he had under observation four years ago. The patient was a young woman of 22, who had been married three years. She had a big swelling on the left side, which on operation proved to be a gray tumor. Dr. Jeffries reported a round-celled sarcoma.

While ill in bed she developed swelling on both sides, and a pelvic tumor near the site of the original tumor. Examination nine months later disclosed an absolutely normal pelvis. Amylopsin and trypsin had been carefully given during this period, and had apparently been effective in producing a cure.

---

### A DISCUSION OF CERTAIN FACTORS RELATIVE TO THE ETIOLOGY OF PELLAGRA.

By C. C. Bass, M. D., New Orleans, La.

Within the past two or three years several new theories as to the cause of pellagra have been advanced, among which may be mentioned, (a) that it is due to some protozoon carried by the similium reptans (Sambon); (b) that it is contagious and due to contact with Italians; (c) that it is due to ingesting cotton seed oil taken in its pure state or in the form of lard substitutes. Though such theories as this and others are not based on sufficient actual evidence and are at once disproven by the facts, they are likely to do incalculable harm by diverting attention from, and disturbing confidence in, the *real* cause or causes of the disease. This in turn results in less stress being laid upon the important part of treatment of the disease, viz., withdrawal of its real cause.

A great industry, corn growing, has no doubt, suffered considerably in this country during the past three or four years, because of the recognition of pellagra here, and the theory that it is caused by eating spoiled corn. Though most medical writings have indeed specified *spoiled* corn, quite a few of the newspaper articles and discussions by laymen have failed to specify or especially to emphasize, this quality. Again, the layman often fails to appreciate that *spoiled* corn signifies infection by and growth of bacteria and development of the resultant toxic substances, sometimes elaborated by such bacterial growth; especially on culture media of very complex composition, such aYs immature corn. As examples of other food sources of disease we may cite the case of milk, which is a very wholesome and nutritious article of diet; but if it becomes infected with typhoid bacilli, it may be the source of typhoid fever. Water, in some form or other, is necessary to maintain life; but if it becomes infected with cholera bacilli, it may produce cholera. Likewise, meat enters into the dietary of the majority of people in all lands; but if it becomes infected with certain bacteria and spoils, it will, if eaten, produce ptomaine poisoning.

Corn is a wholesome and nutritious article of diet; but if it becomes infected and is spoiled by certain bacteria, it may produce pellagra. Depending upon the dose of the toxin and the susceptibility of the individual, the severity of the disease will vary from the mildest to the most fatal, just as a small amount of ptomaine may give rise to mild symptoms while a sufficiently large amount will produce death. It therefore is not *corn,* but *spoiled* corn, that produces pellagra. Just what constitutes spoiled corn, what abteria may elaborate in it this specific toxin, and the nature of the toxin, or toxins, have not been successfully worked out; largely because no disease has been produced in experimental animals that can be identified as pellagra. Corn, spoiled by a variety of bacteria, produces disease and death in experimental animals. Recently I have been able to produce a disease, with skin lesions exactly resembling those of pellagra in man, in a fowl; by feeding corn meal spoiled by inoculation with a pure culture obtained from a supposed source of pellagra. A preliminary note will appear in an early number of the Journal of the American Association. The illustration shows the distribution of the lesions on the legs of the fowl.

There would be no more justice in condemning all corn, or corn meal, as food because *spoiled and infected* corn produces pellagra, than there would be in condemning all milk, because *infected* milk may give rise to typhoid fever. This is true, however, only provided the sources of infection of each, and the infection itself, are equally recognized and controlled. I therefore desire to here point out some of the factors contributing to the spoiling of corn.

It may be laid down as a rule that the susceptibility of corn to bacterial decomposition depends for one very important factor upon its moisture content.

Moisture is one requisite of all bacterial growth. The amount of moisture present in corn will depend upon the manner in which it is grown, harvested, stored, and, to some extent, upon the variety of corn. If grown where the season is short, such as in the northern part of the United States, it may fully mature, and if harvested early will contain much moisture. If, on the other hand, the season is long, as in the Southern States so that the corn fully matures, and if it is allowed to remain in the field until it is well dried, it may be of almost flint-like hardness and very dry. The practice of storing corn in open bins exposes it to the weather while the practice in most of the Southern States of storing it in covered houses after it is well matured and dried, reduces the moisture content to the minimum. The dampness in warehouses often contributes to the spoiling of corn and corn meal. Corn is sold by weight, and it is not always that the seller is scrupulous and sufficiently careful that water does not reach it and increase the weight. When corn meal, containing moisture is shipped over long distances and kept closed in dark, hot cars, the most favorable conditions are present for the development of bacteria, and it may result that corn or corn meal is sound and good for food before shipping; is spoiled and unfit for food upon arrival.

A hard, flinty corn, which may be grown in a long season, can hold much less moisture than the softer varieties that must be grown in the more northern sections, on account of the short seasons. It is a matter of common knowledge among Southern farmers that the immature northern and western grown corn can not be kept for long periods of time in the South during the warm season on account of its little resistance to the corn weevil; whereas the home grown corn is much more resistant.

As a result of the facts pointed out above, corn, or corn meal, when grown or produced where the season is short and shipped over great distances in tight, sometimes damp, hot cars, to warm, damp climates, frequently undergoes more or less decomposition either in transit or after arrival and before being consumed for food. As a matter of act, the chief source of spoiled corn and corn meal in the Southern States is imported corn. If *spoiled* corn is the cause of pellagra, then the chief source of pellagra in the Southern States is imported corn. This is borne out by my observations in Louisiana and in South Mississippi, that the disease here occurs chiefly among the laboring class in country towns and districts, who eat a great deal of corn or orn meal which is generally supplied from the store or ommissary, which, in turn, imports from the corn growing sections. The lack of diversification by the cotton and sugar planters of these two States, compels them to import much of their corn, and with this importation they supply their employees. Much of the pellagra of this section develops among the employees on the cotton and sugar plantations, and especially among the employees on public works and saw mills in the towns and the country; and very frequently among those supplied from the commissary. The point may well be made that pellagra occurs, and in certain instances many cases are found, in the corn belt, for instance, in Peoria, Illinois. To this I would reply that the disease has occurred chiefly in insane hospitals, asylums and other institutions where corn meal and flour are usually supplied by contract and bought and stored in considerable quantities. The supplies for several months or a year may be purchased at the same time. Whole corn does not spoil so rapidly as corn meal. In the Southern States the disease is more generally distributed, I believe; and is not confined to asylums, etc., as is the tendency in other States.

The apparent influence of the cotton boll weevil on the increase and decrease of pellagra in certain Southern States is interesting in this connection. From one to three years ago the States of Louisiana and Mississippi were overrun by the boll weevil and, cotton farming becoming unprofitable, the farmers turned to raising other crops and the yield of corn has been doubled and trebled, and as a result there has been very much less corn or corn meal imported.

In 1909 the type of pellagra diagnosed in New Orleans was very malignant and most of the cases died. In 1910, more cases were diagnosed, it is true, but the mortality was much lower.

The cases diagnosed in 1911, are of a still milder type; and almost all have recovered or will do so. It is possible that as many will be diagnosed here in 1911 as in 1909, and it might at first be thought that the disease is increasing. *Such is not the case.* In 1909, when we first began to recognize the disease only the severe, well-marked cases were diagnosed and most of the cases we are now recognizing would have passed unnoticed. I have no doubt that had we then possessed the same ability to diagnose mild cases, we would have recognized five times as many cases in 1909 as we will in 1911. The first week in October the writer wanted some cases of pellagra for a clinic and after searching the New Orleans Charity Hospital (900 beds), found only one mild case and one doubtful. At any time during the summer and fall of 1909, several well-marked cases could have been selected. This change I attribute largely to the reduction of imported corn and corn meal and the increase of the home supply.

In Eastern Mississippi there is very little reduction as yet in the severity of the disease, and, of course, larger numbers are being diagnosed as the profession becomes more familiar with it. The influence of the boll weevil in its slow spread eastward is not felt to the same extent that it is in a section like Louisiana, which was visited by the pest two or three years earlier. In Georgia or the Carolinas, where the boll weevil has not reached, where the staple crop it cotton and the corn meal supply is largely imported, the disease is thought to be rapidly increasing. It would be interesting to know just how great the increase really is, however, after making due allowance for increased knowledge on the subject gained by the profession.

Briefly referring to the newer theories as to the cause of pellagra, I would point out that simulium does not exist in the southern United States where pellagra prevails to the same extent that it does in other States, like Idaho, *where there is no pellagra.*

With regard to contagiousness due to contact with Italians; I would say that I have studied over three hundred cases of pellagra in the past three years, and *have not seen a sinble case of pellagra in the Italian.* More than half of these cases have come from within a radius of one hundred miles of New Orleans, which section of country contains very many Italian laborers and inhabitants. A good number of the cases have been seen in the New Orleans Charity Hospital where many Italians are treated for other diseases. It would be passing strange if the Italians spread the disease by contact, but at the same time remained free

With regard to the theory recently suggested that pellagra from it themselves.
is due to eating cotton seed oil or lard substitutes made therefrom, it may be set aside with a few words. Pellagra has been known in Egypt, Spain, Italy and Roumania for from twenty-five to one hundred years before cotton seed oil or any of the lard substitutes into which it enters as an ingredient were used for food, Mixell (Journal-Record of Medicine, Vol. LVII), who proposes this theory points to the fact that the increase in the number of cases of pellagra diagnosed in the United States has augmented with the increase of the use of cotton seed oil for food. He could with equal justice accuse, as causative of pellagra, a score or more of other foods, etc., such as canned goods of all sorts, all kinds of breakfast foods, soft drinks and many other things none of which has ever been conceived to have the slightest relation with the disease in question. If the claim of Mixell—that cotton seed oil and its products are responsible for pellagra—is at all tenable, why is it that the use of these food stuffs, which for years have had and are now having enormously increased sale and consumption, in countries like France, Germany, Sweden, Denmark and Turkey, has not been productive of the disease? In the countries referred to, pellagra is little known. Why it is, also, that enormously increased consumption of the products in the larger centers of population in the United States, like New York City, Philadelphia, etc., has not produced the disease? In these centers the disease is practically unknown.

One of the strong points argued by him in support of his new theory, is the fact, well known, of course, to the author, that pigs, or calves, when fed *exclusively* on cotton seed or cotton seed meal, *accumulate fat* and finally die. This is a striking contrast to the actual conditions in pellagra, *in the majority of which emaciation* is extreme before fatal termination. This is no more like pellagra than bloat in cattle, due to eating green clover, is like it.

In conclusion I would emphasize that it is not corn, but *infected, or spoiled* corn that *produces* pellagra;

That corn shipped long distance in tight, dark, damp cars, is more likely to bceome damaged than home grown corn;

That corn grown in section of the country where the season are short, may not fully mature and may therefore be more likely to *spoil* than that grown where the season is longer;

That *sound*, home grown corn is harmless and *is to be commended as food for man.*

I have written freely my opinions, many of which are formed from the study of the literature on pellagra, without referring to the many writings from which the ideas are derived. My indebtedness to these I acknowledge. The literature on pellagra is now too voluminous to permit of just abstraction on the points made in this paper in an article of this length.—The Louisville Monthly Journal of Medicine and Surgery, Nov. 1911.

---

### THE OWENS BILL.

In the Senate of the United States, April 6, 1911, Mr. Owen introduced the folowing bill; which was read twice and referred to the Committee on Public Health and National Quarantine.

To establish a Department of Health, and for other purposes.

*Be it enacted by the Senate and House of Representatives of the United States of America in Congress Assembled.* That there be at the seat of government an executive department known as the Department of Health, and a Director of Health, who shall be the head thereof; and the provisions of title four of the Revised Statutes including all amendments thereto, are hereby made applicable to said department. The Director of Health shall be appointed by the President, by and with the advice and consent of the Senate at a salary of ______ dollars per annum and with tenure of office like that of the heads of the other executive departments. And said director shall cause a seal to be made for the

Department of Health, of such device as the President approves, and judicial notice shall be taken of said seal.

SEC. 2. That there be in the Department of Health an assistant to the Director of Health, designated and known as the Commissioner of Health, who shall be a skilled sanitarian, appointed by the President, by and with the advice and consent of the Senate, who shall serve at the pleasure of the President, and who shall receive a salary of ------ dollars per annum. The Commissioner of Health shall perform such duties as are required by law and such as are prescribed by the Director of Health. There shall be also a chief clerk, a disbursing clerk, and such other employees as Congress may from time to time authorize. The Auditor for the State and Other Departments shall receive and examine all accounts of moneys paid in and of moneys expended on account of the Department of Health, and shall certify the balance arising thereon to the Division of Bookkeeping and Warrants of the Treasury Department, and forthwith send a copy of each such certificate to the Director of Health.

SEC. 3. That it be the province and duty of the Department of Health to foster and promote all matters pertaining to the conservation and improvement of the public health and to collect and disseminate information relating thereto: *Provided,* That this Act shall not be construed as attempting to authorize the Department of Health to exercise or attempt to exercise, without express invitation from the chief executive or other proper authority of the State, any function belonging exclusively to such State, or to enter any premises in any State without the consent of the owner or occupant thereof; but the Director of Health, upon request of the chief executive or other proper authority of any State, Territory, the District of Columbia, or any insular possession, may detail for limited periods an officer or officers, employee or employees, from the Department of Health to assist the health authorities of such State, Territory, District, or insular possession in protecting and promoting the health of the people of such jurisdiction: *And provided further,* That the Department of Health shall recognize no so-called school or system of medicine.

SEC. 4. That to the Department of Health are hereby transferred the following bureaus, divisions, and other branches of the Government, and all that pertains to them, and they and each of them shall remain under the supervision and direction of the Director of Health until otherwise directed by law, namely:

(a) From the Department of the Treasury is transferred the Public Health and Marine-Hospital Service.

(b) From the Department of Agriculture is transferred that part of the Bureau of Chemistry charged with the investigation of the adulteration of foods, drugs, and liquors, and with the execution and enforcement of the Act of Congress entitled "An Act for preventing the manufacture, sale, or transportation of adulterated or misbranded or poisonous or deleterious foods, drugs, medicines, and liquors, and for regulating traffic therein, and for other purposes," approved June thirtieth, nineteen hundred and six.

(c) From the Department of Commerce and Labor is transferred the Division of Vital Statistics, Bureau of the Census.

And the President is hereby authorized to transfer to the Department of Health at any time either the whole or any part, as to him may seem best, of any bureau, division or other branch of the Government engaged in work pertaining to the public health, except the Medical Department of the Army and the Bureau of Medicine and Surgery of the Navy.

And each and every function, authority, power, duty and jurisdiction, of whatsoever character it may be, vested at the time of any transfer aforesaid in the head of the executive department from which such bureau, division, or other branch of the Government is transferred, shall, to the extent of which such function, authority, power, duty or jurisdiction pertains to such bureau, division, or other branch of the Government, immediately upon such transfer become vested and thereafter remain vested in the Director of Health.

All land, buildings, furniture, apparatus, equipment, and property of whatever description, and all official records and papers in the custody of any executive department from which any bureau, division, or other branch of the Government is transferred as aforesaid and pertaining to the business of such transferred bureau, division, or other branch of the Government, shall at the time of such transfer, or as soon thereafter as practicable, and in so far as such action can be taken without hindering the work of the executive department from which such transfer is made, be given over into the custody of the Department of Health. And all unexpended balances of appropriations available at the time of such transfer for the use of any such transferred bureau, divison or other branch of the Government, or which may become available thereafter, shall be and remain available, in similar manner and to the same extent as if no transfer had been made.

SEC. 5. That within the Department of Health there shall be the following bureaus:

(a) Bureau of Sanitary Research; (b) Bureau of Child Hygiene; (c) Bureau of Vital Statistics and Publications; (d) Bureau of Foods and Drugs; (e) Bureau of Quarantine; (f) Bureau of Sanitary Engineering; (g) Bureau of Government Hospitals; (h) Bureau of Personnel and Accounts. And the Director of Health is hereby authorized to arrange and rearrange from time to time, with the approval of the President, the functions, duties, personnel, papers, records, and property, and the work, resources, and equipment generally, coming into the jurisdiction and control of the Department of Health by the operation of this Act, so as most efficiently and economically to organize and maintain the several bureaus herein named and such divisions and offices thereof as to said director seems proper; but in arranging and rearranging the personnel, the rank, pay, and allowances of the officers of the Public Health and Marine-Hospital Service commissioned at the time of the transfer of that service to the Department of Health shall not by reason of anything in this Act contained, be diminished. And the Director of Health may call upon the heads of other executive departments for information in their possession, whenever such information is needed for the efficient and economical working of the Department of Health.

SEC. 6. That the President is hereby authorized to detail officers and employees from any of the several executive departments of the Government for duty under the Director of Health when so requested by said director and to detail officers and employees in the service of the Department of Health to any of the other executive departments upon request of the head of such department, provided such detail can be made without prejudice to the public service, to carry into effect the purpose and intent of this Act; but officers and employees so detailed shall receive no additional compensation, but shall be paid such actual and necessary expenses as they incur in the discharge of their duties.

SEC. 7. That the Director of Health may, in his discretion and with the approval of the President, appoint an advisory Board of not more than seven members, to confer with him upon his request, from time to time as he deems necessary, concerning the work of the Department of Health and the health of the people. The members of said board shall be selected because of their special knowledge of matters relating to the public health, and each shall hold office for a term of seven years or until his successor is appointed, except that the appointments first made, and appointments thereafter made to fill unexpired terms and terms of members who have held over beyond the periods of their original appointments, shall be made so that not more than one member shall retire during any one fiscal year. No member of any such advisory board shall receive any compensation for his services, but each shall be paid all actual expenses necessarily incurred in the discharge of his duties. And from and after the passage of this Act the advisory board for the Hygienic Laboratory created by section five of an Act entitled "An Act to increase the efficiency and change the name of the United States Marine-Hospital Service," approved July first, nineteen hundred and two, be, and the same hereby is, abolished.

SEC. 8. That the Director of Health may, whenever in his judgment public interests would be promoted by so doing, invite the duly constituted health authorities of all or of any of the States, Territories, the District of Columbia, and insular possessions as to him may seem advisable, each to send one delegate to confer with him or his duly appointed representative or representatives and with each other, at such time and place as he may designate, concerning any particular matter or matters relating to the public health; and it shall be the duty of the Director of Health, upon the written application of the duly constituted health authorities of not less than five States, Territories, the District of Columbia, and insular possession, stating the particular matter or matters which it is desirable to consider, to appoint a time and place, and to call a conference of the health authorities of the States, Territories, the District of Columbia and insular possessions that united in the request therefor, and personally or through his duly appointed representative or representatives to be present at such conference; but every State, Territory, the District of Columbia, and insular possession shall be notified of every conference, and if practicable, be afforded an opportunity of being present and participating in its proceedings. And from and after the passage of this Act annual and other conferences of State and Territorial boards of health, quarantine authorities, and State health officers, provided for by section seven of an Act entitled "An Act to increase the efficiency and change the name of the United States Marine-Hospital Service," approved July first, nineteen hundred and two, be, and the same are hereby, abolished.

SEC. 9. That, except as expressly provided in this Act, nothing shall be construed as limiting or abrogating any function, right, or duty imposed by law upon any existing bureau, division, or other branch of the Government, but such bureaus, divisions, and other branches of the Government as are by this Act or by authority thereof transferred to the Department of Health shall continue, under direction of the Director of Health, to have such functions, duties, and rights as they have at the time of such transfer; and in the case of such bureaus, divisions, and other agencies of the Government as are transferred in part only, the part not transferred shall continue to have and to exercise all such functions, duties, and rights, except such as specifically relate to the part transferred to the Department of Health, in the same manner and to the same extent as if no such transfer had been made.

SEC. 10. That the Director of Health shall annually submit to Congress a report in writing showing the operations of the Department of Health during the last preceding fiscal year, which report shall give an account of all moneys received and all moneys disbursed on account of such operations. He shall make such other reports from time to time as may be required by the President, or by either House of Congress, and such as are in his judgment necessary or expedient.

SEC. 11. That ______ dollars be, and the same are hereby, appropriated to carry into effect the provisions of this Act, out of any of the money in the Treasury not otherwise appropriated.

SEC. 12. That all Acts and parts of Acts contrary to the provisions of this Act or inconsistent therewith, be and the same are hereby, repealed.

SEC. 13. That this Act shall take effect on and after July first, nineteen hundred and twelve.

---

### THE PRIVATE PHYSICIAN AND PUBLIC SANITATION.

The spirit of prophylaxis pervades the air. On all sides we see the hopeful tendency increasing to preenvt the evil day of disease and suffering rather than to calmly await its arrival, and then blindly fight the dire results of our neglect and ignorance.

Realizing the fact that all sound sanitation must rest upon a basis of uniformity in vital statistics, the Census Bureau has recently compiled and issued free to all registered physicians in the United States a handbook of the international list of the causes of death. This little leaflet is so inconspicuous in appearance and its container so reminds one of an advertisement that we greatly fear many of our readers have consigned it to the waste-basket without opening the envelope in which it came and, therefore, remain in blissful ignorance of its great value. If perchance such a one may be the reader of these lines we exhort him to remedy his oversight at once by requesting a copy from the chiefs of the Census Bureau. We believe that the general practitioner is not sufficiently alert as to the great responsibility that rests upon his shoulders in the matter of the simple recording of the facts of birth and the causes of death which come under his observation. Take, for instance, the simple question of nomenclature, and let it be illustrated by the confusion which exists in such an important disease as acute anterior poliomyelitis. During 1909 this disease was returned from the different registration areas of the United States under no less than twenty-four separate headings. How can the important clinical data absolutely necessary to the proper interpretation of the findings of the laboratory men ever be successfully collected when the general practitioner shows such indifference to the use of exact terms? Apropos both of this particular subject and the general one of prophylaxis, we are glad to note that the Commissioner of Health of the State of New York called a conference of the State department health officers at Albany on July 3 to consider the field methods of investigation to be undertaken in the event of a recurrence of an epidemic of acute anterior poliomyelitis during this summer and fall. The Rockefeller Institute has served notice to the profession at large that it will be willing to receive and treat gratis any such cases arising in the practice of physicians, and has asked that in the event of the death of any such cases where an autopsy has been made, that specimens of portions of the spinal cord, of the nasal mucous membrane, tonsils and adenoids be preserved in glycerin and forwarded to Dr. Flexner. Considering the fact that only nine cases of acute anterior poliomyelitis were reported from the whole State of New York for the month of June, we are more than ever impressed with the cool scientific deliberateness of the Rockefeller Institute and the New York State Department of Health. Surely, this is no spasmodic effort to lock the stable after the horse is stolen. Now, let physicians from all over the United States help the work along and share in the glory of the solution of the problem by sending the specimens desired whenever an opportunity presents, not forgetting to forward a complete history of the case in all its environmental relations.

But it is not State organizations and privately endowed institutions alone that are forwarding this general movement of prophylaxis. The federal services with even less hope of pecuniary reward or special honor are at the very forefront of all movements looking towards the conservation of the public health. Taking time by the forelock, and profiting by the experience of the Spanish-American War and the lessons learned in the Russian-Japanese conflict, the medical corps of the United States Army is leaving no stone unturned to provide against the contingencies of war. With this end in view, a series of manuals, dealing with the duties of the medical officers in the field of service in time of war are being prepared and issued to all physicians in the medical reserve corps. These little booklets are so handsomely bound, and so graphically written that we fancy there is not one of their recipients who has not read them with interest and profit, and who does not cherish them as valuable additions to his library. The experimental work in anti-typhoid vaccination which the army has conducted for the last three years has borne adequate fruit in the mobilization of the troops on the Mexican border. There, among an army of 20,000 troops, camped in most unfavorable conditions of rain and mud, scarcely a case of typhoid developed. What a contrast to the days of Camp Chickamauga and what a lesson in the value of preventive medicine!

Reading the possibility of the future in the dire calamity of the past when the plague left London desolate, and realizing that the present situation in Manchuria shows the possibility of history repeating itself in the West, the United States Public Health and Marine Hospital Service carries on unremittingly its work of rat and squirrel destruction in all those areas where rodent plague has been demonstrated. Were it not for this work of prophylaxis, there is no reason to believe that our western coast would not be almost as plague-ridden as are India and China.

Even the field of the alienist is being invaded by the sanitarium, and the air is filled with the exhortations of the disciples of Galton, teaching the possibilities of the betterment of the race by the study of eugenics.

Through all this agitation and hue and cry to prevent, there runs one discordant note. The time is ripe for the unification of all the various private institutions for research and the different State and federal health autorities under one general head.

In the meantime, and while awaiting the coming of that happy day, the general practitioner has one great duty to perform. He must get himself in training for the performance of public service and, his first step in this direction should be to aid the movement for universal sanitation by the careful recording of the vital statistics of his own private practice.—Ed. Lancet Clinic.

